# A Comprehensive Study on the Influence of Superheated Steam Treatment on Lipolytic Enzymes, Physicochemical Characteristics, and Volatile Composition of Lightly Milled Rice

**DOI:** 10.3390/foods13020240

**Published:** 2024-01-11

**Authors:** Chenguang Zhou, Bin Li, Wenli Yang, Tianrui Liu, Haoran Yu, Siyao Liu, Zhen Yang

**Affiliations:** 1 Agricultural Product Processing and Storage Lab, School of Food and Biological Engineering, Jiangsu University, Zhenjiang 212013, China; 2 School of Pharmacy, Jiangsu University, Zhenjiang 212013, China; 3 Key Laboratory of Nuclear Agricultural Sciences of Ministry of Agriculture and Zhejiang Province, Institute of Nuclear Agricultural Sciences, Zhejiang University, Hangzhou 310058, China

**Keywords:** superheated steam, rice, lipolytic enzymes, GC/MS

## Abstract

Enzyme inactivation is crucial for enhancing the shelf life of lightly milled rice (LMR), yet the impact of diverse superheated steam (SS) treatment conditions on lipolytic enzyme efficiency, physicochemical properties, and volatile profiles of LMR remains unclear. This study investigated varying SS conditions, employing temperatures of 120 °C, 140 °C, and 160 °C and exposure times of 2, 4, 6, and 8 min. The research aimed to discern the influence of these conditions on enzyme activities, physicochemical characteristics, and quality attributes of LMR. Results indicated a significant rise in the inactivation rate with increased treatment temperature or duration, achieving a notable 70% reduction in enzyme activities at 120 °C for 6 min. Prolonged exposure to higher temperatures also induced pronounced fissures on LMR surfaces. Furthermore, intensive SS treatment led to a noteworthy 5.52% reduction in the relative crystallinity of LMR starch. GC/MS analysis revealed a consequential decrease, ranging from 44.7% to 65.7%, in undesirable odor ketones post-SS treatment. These findings underscore the potential of SS treatment in enhancing the commercial attributes of LMR.

## 1. Introduction

Rice (*Oryza sativa*) stands as a primary global crop and serves as the staple food for over half of the world’s population. The rice crop undergoes husking to yield brown rice, which, in turn, is further milled to eliminate the bran layer and germ, resulting in white rice—the prevalent form of consumption. In contrast to white rice (WR), brown rice (BR) harbors enhanced bioactive constituents, including lipids, amino acids, vitamins, phytosterols, and phenolic compounds, attributable to the presence of bran and embryo [1]. Despite its nutritional richness, BR undergoes limited consumption as a staple due to prolonged cooking times, firm texture, and the undesirable odor associated with rice bran. Lightly milled rice (LMR), obtained by selectively removing the bran layer while retaining the embryo during the milling process, has emerged as a favored rice variant. Recent investigations demonstrate that this method of light milling substantially enhances cooking quality compared to brown rice while preserving a significant portion of its nutritional contents [2].

The milling process for LMR, however, may compromise the natural barrier between enriched lipids and lipolytic enzymes in the bran layer. This leads to accelerated hydrolytic rancidity and oxidative rancidity of lipids, causing LMR to have a shorter shelf life than BR and WR. Consequently, the inactivation of these enzymes becomes crucial for the extended storage of LMR. While heat treatments are commonly employed for enzyme inactivation in foods, traditional approaches like high-temperature air fluidization [3] and microwave [4] and infrared [5] treatments often alter the natural physicochemical properties of foods, resulting in nutrient loss or fissure formation, significantly impacting the commercial quality of rice.

Superheated steam (SS) emerges as a promising heat treatment technology suitable for various food processing applications. Its primary advantages lie in high thermal penetration and the provision of an oxygen-free environment, leading to swift heating and reduced oxidative degradation reactions during processing. Consequently, the utility of SS as a heat treatment medium has garnered increasing attention. In recent years, SS has found widespread use in enzyme inactivation to prolong the shelf life of grains. For instance, Wang et al. [6] demonstrated that treating buckwheat grains with SS at 170 °C for 5 min significantly inhibited lipase activity, suppressing hydrolytic rancidity. Similarly, the treatment of black soybeans with SS at 190 °C for 40 s proved effective in inactivating lipolytic enzymes, thereby enhancing lipid stability [7]. Numerous studies have also reported a substantial reduction in residual enzyme activities following SS treatment [8,9,10].

Nevertheless, it should be noted that LMR exhibits characteristics distinct from those of whole grains due to the absence of the protective outer layer. In the absence of the cuticular layer, LMR is susceptible to developing undesired cracks during SS processing. However, there is a scarcity of comprehensive studies addressing the impacts of superheated steam (SS) treatment on lightly milled grains. The previous literature underscored the significance of SS treatment parameters, particularly temperature and duration, in influencing enzyme inactivation and various physicochemical properties. The primary objective of this study is to systematically evaluate the influence of different SS treatment conditions on LMR enzyme activities. Furthermore, the study aims to comprehensively assess the impact of varying SS conditions on the physicochemical and quality characteristics of LMR.

## 2. Materials and Methods

### 2.1. Chemicals and Reagents

The internal standard 4-methyl-2-pentanol was supplied by Macklin Inc. (Shanghai, China). The n-Alkane mix (C7–C40) was purchased from Sigma-Aldrich (Steinheim, Germany). Other chemicals were procured from Sinopharm Chemical Reagent Company (Shanghai, China).

### 2.2. Design of Experiment and Procedure

Vacuum-packed brown rice was procured from Dandong, Liaoning Province, and then milled with a rice milling machine (Model JM3010, Shenzhen Mifresh Technology Com., Ltd., Shenzhen, China) to obtain LMR. The LMR was then subjected to treatment using SS equipment (Model LHCCK2, Nanjing Leying Professional Kitchen Equipment Co., Ltd., Nanjing, China) under the following conditions: the temperature was adjusted to 120 °C, 140 °C, and 160 °C, and the volume flow was maintained at 0.5 m^3^/min. The treatment time was varied between 0, 2, 4, 6, and 8 min. Once the SS reached the predetermined temperature, 50 g of LMR was evenly spread in a single layer onto the sieve and fed into the processing chamber via a conveyor belt, ensuring uniform passage of SS through the LMR. The processed samples were then sealed and refrigerated at 4 °C for later use.

### 2.3. Observed Response in LMR

#### 2.3.1. Moisture Content and Distribution

The moisture content of the LMR samples was analyzed using a moisture analyzer (Model Hr83, Mettler toledo Inc., Zurich, Switzerland). The moisture distribution was determined by a low-field nuclear magnetic resonance (LF-NMR) analyzer (NMI20-060V-I, Niumag Electric Co., Ltd., Shanghai, China). The transverse relaxation (T2) of the samples was measured at a resonance frequency of 22 MHz and a temperature of 32 °C using the Carr–Purcell–Meiboom–Gill sequence. The optimal parameters for T2 measurement were established as follows: spectral width (SW) = 200 KHz, repeated sampling waiting time (TW) = 2600 ms, spectrometer frequency (SF) = 21 MHz, radio frequency delay (RFD) = 0.002 ms, 90° pulse time (P1) = 5 μs, 180° pulse time (P2) = 13 μs, echo time (TE) = 0.2 ms, number of data points (TD) = 1024, number of accumulative sampling times (NS) = 4, analog gain (RG1) = 20 db, and digital gain (DRG1) = 3.

#### 2.3.2. Enzyme Activity

To prepare the sample for enzyme activity determination, the untreated and treated LMR were ground into a fine powder using a high-speed universal crusher (Model LG 50, Ruian Baixin Pharmaceutical Machinery Co., Ltd., Ruian, China) and sieved through a 180 μm standard sieve.

##### Lipase Activity Determination

Lipase activity was determined using the method described by Rose and Pike [11]. Specifically, 2 g of LMR was extracted with 20 mL of hexane in a 50 mL screwcap tube for 30 min on a shaker (Model HYL-C, QiangLe, Wenzhou, China) at 140 rpm. The mixture was then centrifuged (Model TGL-15B, Boyikang, Beijing, China) at 8000× *g* for 5 min, and the supernatant was discarded. Next, 4 mL of 50% (*w*/*v*) olive oil in hexane was introduced to the defatted sample, and the tube was subjected to incubation and oscillation in a water bath at 40 °C for 4 h. Upon completion of the incubation, the hexane was evaporated using a speed vac, and the resulting residue was redissolved in 10 mL of isooctane to ensure complete extraction of free fatty acids (FFA).

The quantification of FFA followed the protocol of Goffman and Bergman [12]. Specifically, 1 mL of 3% (*v*/*v*) pyridine in 5% (*w*/*v*) aqueous cupric acetate was added to the FFA sample, followed by shaking at 250 rpm for 5 min to facilitate color development. The tubes were centrifuged at 5000× *g* for 5 min, and the resulting supernatant was collected and analyzed for absorbance at 715 nm using a spectrophotometer (Model UV1601, Rayleigh, Shanghai, China). Lipase activity was quantified based on an external standard curve of oleic acid (C18:1) and expressed as U/g LMR, where 1 U/g = 1 µmol C18:1 eq/h/g LMR.

##### Lipoxygenase Activity Determination

Lipoxygenase activity was assayed according to the method described by Mohammadi et al. [13]. Specifically, 1 g of LMR was mixed with 2.4 mL of potassium phosphate buffer (pH 7.4), and the resulting slurry was centrifuged at 9000× *g* for 15 min to yield the supernatant as the enzyme source. For substrate solution preparation, 0.5 mL of Tween 20 was dissolved in 10 mL of borate buffer (pH 9.0), followed by slow addition of 0.5 mL of linoleic acid and subsequent addition of 1.3 mL of NaOH to achieve clarity. The final volume of substrate was adjusted to 200 mL with distilled water after the addition of 90 mL of borate buffer. The reaction mixture consisted of 2.0 mL of potassium phosphate buffer, 500 μL of the extracted enzyme, and 500 μL of substrate. The absorbance of the mixture was measured using a spectrophotometer at 234 nm, and one unit of lipoxygenase activity was defined as the changes in absorbance of 0.001/min under the specified experimental conditions over a 3 min reaction period.

##### Peroxidase Activity Determination

The peroxidase activity was assessed following the method described by Jiang et al. [14]. In brief, 1 g of LMR was mixed with 10 mL of potassium phosphate buffer (pH 7.4) and then centrifuged at 5000× *g* for 10 min. The resulting supernatant was collected as the source of enzyme. For substrate preparation, 0.5 mL of guaiacol and 0.5 mL of hydrogen peroxide were mixed with 99 mL of sodium phosphate buffer (pH 6.0). The reaction mixture consisted of 1 mL of enzyme extract and 3 mL of substrate solution, and the absorbance was recorded at 470 nm for 3 min using a spectrophotometer. One unit of peroxidase activity was defined as the changes in absorbance of 0.001/min under the assay conditions.

#### 2.3.3. Grain Morphology

##### Color Determination

The color of untreated and SS-treated LMR grains was determined by means of a digital chroma meter (CM-2300d, Konica Minolta, Tokyo, Japan) and expressed as L* (lightness/darkness), a* (greenness/redness), and b* (blueness/yellowness) values. The color difference (ΔE) was calculated using the following formula:

ΔE* = [(L* − L_0_*)^2^ + (a* − a_0_*)^2^ + (b* − b_0_*)2]^1/2^,

where the subscript “0” refers to the chromaticity values of the untreated LMR.

##### Grain Morphology Assessment

The morphological characteristics of the LMR samples were examined under a stereomicroscope (SZM, SUNNY, Yuyao, China) with lateral illumination. The images captured with the stereomicroscope were exported using the MvImage software (version 1.0.5, SUNNY, China).

#### 2.3.4. Physicochemical Properties of LMR Starch

##### Starch Extraction

Starch was extracted from LMR following the method described by Li et al. [15]. Initially, 10 g of LMR was soaked in 30 mL of water at 26 °C for 3 h, milled, and then centrifuged at 5000× *g* for 5 min. The resulting rice sediment was dried at 40 °C for 8 h to obtain dried rice flour. The rice flour was subsequently mixed with 30 mL of 0.2% sodium hydroxide solution and stirred for 12 h to facilitate the removal of protein. The rice slurry was centrifuged at 3000× *g* for 15 min, and the supernatant was discarded. The sediment was resuspended in 30 mL of water, followed by neutralization using hydrochloric acid solution. The neutralized slurry was centrifuged, and the sediment was washed with 30 mL of water three times. The generated starch was freeze-dried, passed through a 200-mesh sieve, and stored for further analysis.

##### X-ray Diffraction (XRD)

The crystalline structure of the LMR starch was analyzed using an X-ray diffractometer (Model D8 ADVANCE, BRUKER, Ettlingen, Germany) in accordance with the method described by Ren et al. [16]. The XRD patterns were obtained using a voltage of 40 kV and a current of 40 mA. The scanning range was from 5° to 40° (2θ), with a scanning speed of 5°/min and a step size of 0.02°. The relative crystallinity was determined using JADE software 5.0 (Materials Data Inc., Livermore, CA, USA).

##### Fourier Transform Infrared Spectroscopy (FTIR)

The Fourier transform infrared (FTIR) spectra of starch samples were collected using a Nicolet IS50 spectrometer (Thermo Nicolet Corporation, Waltham, MA, USA) equipped with a universal attenuated total reflectance (ATR) accessory, following the protocol described by Huang et al. [17]. The spectra were scanned over the range of 4000 to 400 cm^−1^, with a resolution of 4 cm^−1^ and an accumulation of 64 scans. Subsequently, all spectra were automatically baseline-corrected using OMNIC 8.0 software. The spectra from 1200 to 800 cm^−1^ were deconvolved with a half-bandwidth of 19 cm^−1^ and an enhancement factor of 1.9. The absorbance ratios at 1047/1022 cm^−1^ and 995/1022 cm^−1^ were calculated to estimate the short-range ordered structure of starch.

#### 2.3.5. Analysis of Volatile Organic Compounds (VOCs)

Volatile organic compounds were analyzed using gas chromatography–mass spectrometry (GC-MS) (TQ8040, Shimadzu, Japan) [18]. To this end, 5 g of samples and 20 μL of 4-methyl-2-pentanol (at a concentration of 8.02 ng/μL in hexane) as an internal standard were added into a 20 mL headspace glass vial. The DVB/CAR/PDMS 50/30 μm fiber was used to extract volatile compounds for 30 min at 50 °C. The fiber was then thermally desorbed in the GC injector port at 250 °C for 4 min. Separation of the compounds was achieved using a DB-WAX column (30 m × 0.25 mm × 0.25 μm, Agilent Technologies, Santa Clara, CA, USA) [19]. The compounds were identified by matching the mass spectra with the NIST 17 mass spectral library and the Kovats’ retention index (RI) (calculated from C7 to C40 alkanes) with the NIST Chemistry WebBook database https://webbook.nist.gov/chemistry/name-ser/ (accessed on 8 March 2023). The relative contents of volatile compounds were calculated based on the internal standard method.

### 2.4. Statistical Analysis

All experiments were performed in triplicate and the results were presented as means ± standard deviations (SD). To determine significant differences, ANOVA with Tukey’s post hoc test (*p* < 0.05) was performed using XLSTAT (version 19.5, Addinsoft, Paris, France). The multivariate analysis of the volatile data matrices was imported into the online platform Metaboanalyst https://www.metaboanalyst.ca/ (accessed on 23 March 2023). The data were pretreated by applying logarithmic transformation and Pareto scaling to achieve standardization and normalization, respectively.

## 3. Results and Discussion

### 3.1. Effects of SS Treatment on Moisture Content and Distribution

**Figure 1:**
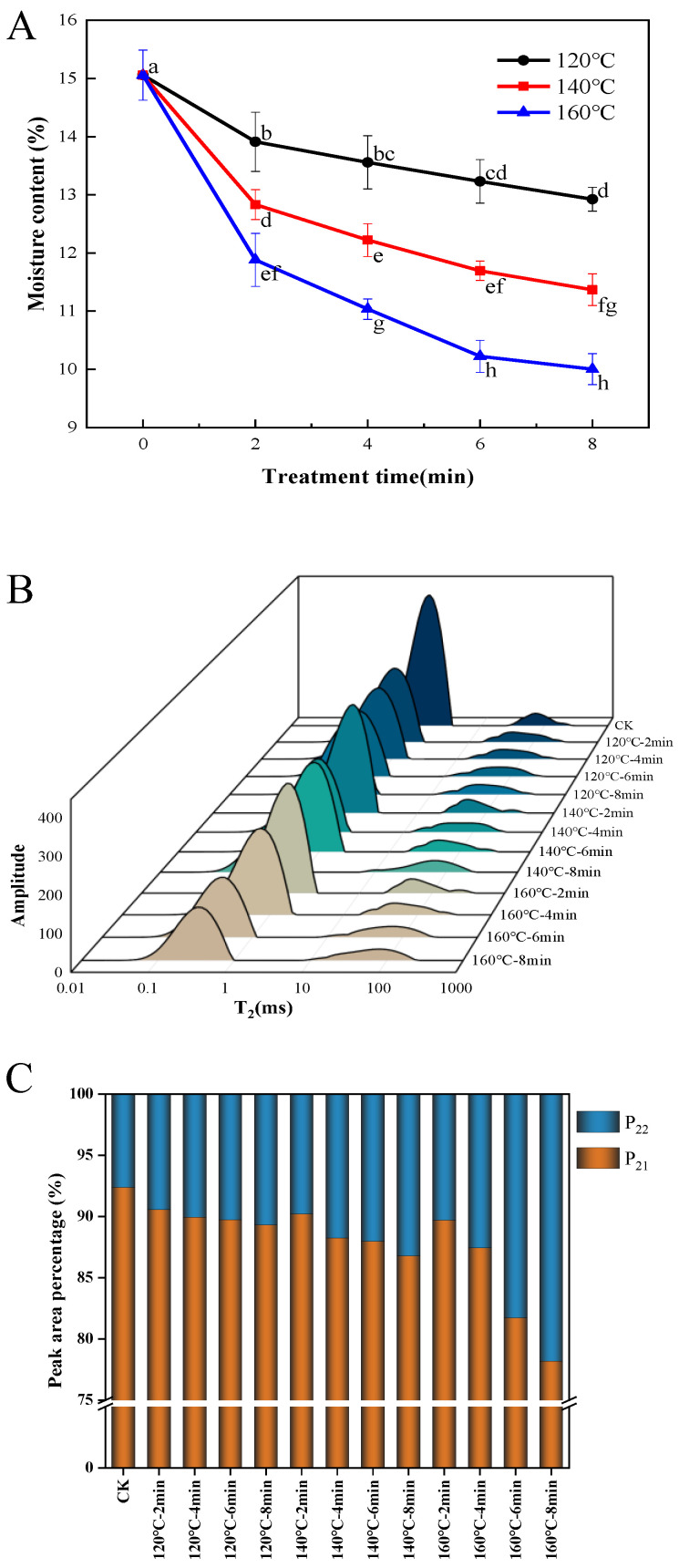
Effect of SS treatment on moisture content (**A**), T2 relaxation time distribution (**B**), and T2 peak ratio (**C**) of LMR. Different letters in the (**A**) indicate a significant difference (*p* < 0.05).

Figure 1A depicts the influence of SS processing temperature and duration on the moisture content of LMR grains. Notably, the moisture content of SS-treated LMR exhibited a diminishing trend with prolonged time and increased temperature, with the most rapid reduction occurring within the initial 2 min. Specifically, when LMR grains underwent treatment at temperatures of 120 °C, 140 °C, and 160 °C for 8 min, their moisture contents decreased from 15.06% to 12.9%, 11.4%, and 10.0%, respectively. Optimal drying conditions are pivotal in preserving the quality of rice during storage. In this investigation, under the mild treatment condition of 120 °C for 4 and 6 min, the moisture content decreased from 15.06% to 13.5% and 13.2%, respectively. Typically, the recommended moisture content threshold for cereal grains for storage falls below 13–14% [20]. Consequently, LMR grains treated at 120 °C for 4 to 6 min could be stored without necessitating additional drying or moisture adjustments. While higher temperatures accelerated the reduction in moisture content (Figure 1A), moderate tempering could mitigate the degradation of bioactive compounds and microstructure induced by prolonged high temperatures [21]. This approach could, in turn, facilitate the preservation of taste, texture, and a low proportion of broken rice [22].

The distribution of water curves in LMR subsequent to superheated steam treatment was assessed based on transverse relaxation time (T2) utilizing LF-NMR. Two distinct water distributions were identified, as depicted in Figure 1B, T_21_ (0.1–10 ms) and T_22_ (10–1000 ms), signifying bound water and free water, respectively [21]. The percentage of the total area within distinct T_2_ intervals serves as an indicator of the relative concentration of H protons in each interval, designated as P_2_ and graphically depicted in Figure 1C. The results underscore that the summit area corresponding to T_21_ was the most pronounced, indicating a superior presence of bound water content in LMR. The investigation revealed that the P_21_ of untreated LMR constituted 92.39% of the total peak area. Moreover, heightened SS treatment intensity resulted in a reduction in the P_21_ proportion, primarily attributed to the significant decrease in T_21_ rather than alterations in T_22_. This finding is in concordance with earlier observations suggesting that SS treatment exerts a more pronounced effect on bound water in grains characterized by low moisture content, with a comparatively minor impact on free water [23]. Additionally, the leftward shift in the T_21_ peak implies that SS steam treatment affects water migration in LMR, particularly concerning bound water.

### 3.2. Effect of SS Treatment on Lipase, LOX, and POD Inactivation

**Figure 2:**
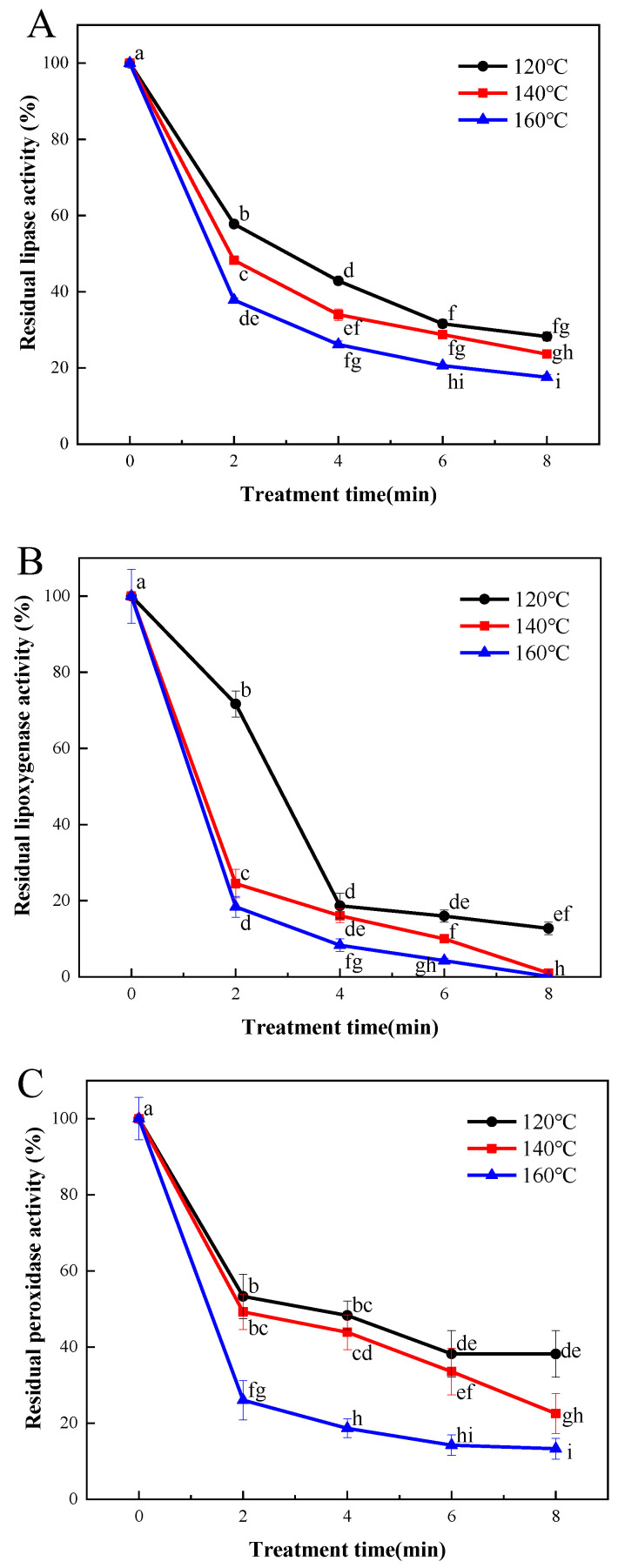
Effect of SS treatment on residual activities of lipase (**A**), lipoxygenase (**B**), and peroxidase (**C**) in LMR. Different letters in the figure indicate a significant difference (*p* < 0.05).

Enzymatic hydrolysis and oxidation, catalyzed by lipase and lipoxygenase, may contribute to the occurrence of rancidity and the generation of off-flavors in cereals. This, in turn, could shorten the shelf life of cereal products. As illustrated in Figure 2A, the efficacy of lipase inactivation in LMR exhibited a strong correlation with the duration and temperature of the SS processing. A reduction of 43.6% in lipase activity was observed in samples subjected to SS treatment at 120 °C for 2 min. Subsequent prolongation of the SS processing time to 4, 6, and 8 min resulted in more substantial decreases in lipase activities, reaching 58.9%, 70.5%, and 71.7%, respectively. A similar declining pattern was observed in SS-treated samples at 140 °C and 160 °C, with final reduction degrees of lipase activities reaching 77.1% and 81.8%, respectively (Figure 2A). It is evident that SS treatment effectively inactivates lipase activity, and the inactivation rates vary considerably among different cereals. For instance, previous research reported a lipase activity reduction of approximately 30% in buckwheat grains after SS processing at 140 °C for 5 min [6]. In contrast, our findings show an approximate 70% reduction in lipase activity for LMR under comparable SS treatment conditions. Similar results were also documented for brown rice [24] and black glutinous rice [25], suggesting the significantly superior heat transfer efficiency of rice grains compared to that of wheat grains [26].

Figure 2B depicts a sharp decline in LOX activity (by 81.3%) upon SS treatment, and this reduction was comparatively more pronounced than that observed for lipase (Figure 2A). These findings align with those of Xu et al. [27], who demonstrated that lipase activity was less heat-labile than lipoxygenase in wheat germs. Nonetheless, Poudel and Rose reported that the lipase activity of whole wheat flour decreased more rapidly than that of lipoxygenase following the steam treatment of wheat grains [28]. Thus, the thermal resistance of lipase and lipoxygenase may vary depending on the specific heat treatment methods employed and the duration of treatment.

Peroxidase (POD) plays a pivotal role in promoting oxidative rancidity, particularly in the case of polyunsaturated fatty acids facilitated by lipase, thereby expediting undesirable chemical changes. Previous research has demonstrated the superior thermal stability of POD compared to lipase and LOX, making it a commonly employed benchmark for determining the optimal thermal treatment necessary for enzyme inactivation [29]. In the current investigation, the activity of POD in LMR exhibited a gradual decline following treatment at 120 °C for 2, 4, 6, and 8 min, resulting in reductions of 46.7%, 51.7%, 61.8%, and 66.0%, respectively (Figure 2C). However, increasing the SS temperature to 140 °C did not yield significant improvements in inactivation effectiveness. In contrast, a substantial reduction of 86.7% in POD activity was achieved when LMR was subjected to treatment at 180 °C for 8 min. These findings are consistent with those reported by Guo et al. [9,28], who demonstrated that the inactivation of POD proved more challenging than that of lipase and lipoxygenase under comparable SS treatment conditions.

### 3.3. Effects of SS Treatment on Color and Surface Morphological Properties of LMR

Rice color constitutes a vital attribute influencing consumer perception and product acceptance. The application of SS treatment induced alterations in the color parameters of LMR, as outlined in Table 1. The control sample exhibited L* (lightness), a* (redness), and b* (yellowness) values of 67.28, 2.10, and 24.47 (Table 1), respectively, signifying the baseline color attributes of LMR. Initial exposure to SS at 120 °C for 2 min yielded no discernible changes in LMR coloration. Notably, the most pronounced increase in L* value (73.05) was recorded in LMR kernels subjected to SS treatment at 160 °C for 8 min, followed by those treated under conditions of 160 °C for 6 min (72.38), 140 °C for 8 min (71.93), and 120 °C for 8 min (71.73). This enhanced lightness can be attributed to the puffing effect on rice kernels, leading to the exposure of the inner white starchy endosperm [30]. This phenomenon displayed a positive correlation with the degree of kernel puffing (R = 0.76, *p* < 0.05). Likewise, incremental SS temperature and prolonged exposure periods yielded slight elevations in a* values (Table 1). Given that SS processing is predominantly an oxygen-free process (except during the initial phase), it is reasonable to surmise that the Maillard reaction, rather than enzymatic reactions, is the principal driver behind these color variations [31,32]. In support of this, Jittanit et al. [33] reported that condensed water from SS could facilitate the migration of reducing sugars and amino acids, both essential components for Maillard reactions.

**Figure 3:**
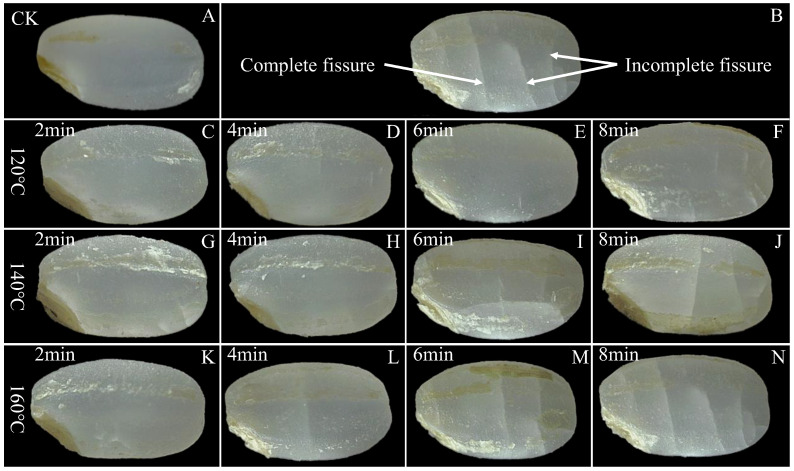
Surface morphology of untreated and SS-treated LMR at different temperatures and durations. An intact and smooth texture of LMR was still maintained upon SS treatment at 120 °C for 6 min (**E**). (**A**): CK (untreatment LMR), (**B**): a diagram of complete and incomplete fissures, (**C**–**N**): LMR treated with SS at 120–160 °C for 2–8 min.

The commercial value of rice is compromised when alterations in grain appearance are identified as indicative of grain damage. Figure 3 provides a visual comparison of the control group and SS-treated LMR under different processing conditions. The surface of untreated LMR exhibits an intact and smooth texture, with no discernible fissures evident after treatment at 120 °C (Figure 3C–F). Upon elevating the temperature to 140 °C, fissures become visible on the surface after 6 min of SS exposure (Figure 3I). Notably, the application of higher temperatures (160 °C) and/or longer durations (6 and 8 min) of SS treatment led to the development of deeper fissures (Figure 3J,M,N). Previous studies have substantiated the physically destructive effects of various thermal treatment techniques on the appearance of cereal grains [30,34,35]. These morphological changes can be attributed to the moisture gradient between the interior and exterior of the LMR grains [3]. Specifically, when LMR is exposed to elevated temperatures, it experiences a phenomenon referred to as “flash-off” of internal liquid water, leading to a rapid evaporation process and the consequent build-up of pressure within the grains, ultimately resulting in their expansion [36]. The resultant high surface tension and compressive stress appear to breach the integrity of the kernel layer, consequently giving rise to the formation of water- and heat-permeable fissures. These fissures, in turn, facilitate mass and heat transfer during SS treatment [3,37].

### 3.4. Effects of SS Treatment on Starch Structure

#### 3.4.1. Long-Range Ordered Structure of Starch

**Figure 4:**
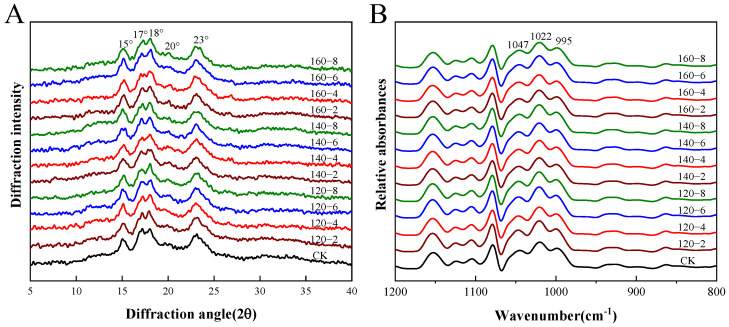
The XRD diffraction patterns (**A**) and FTIR spectra (**B**) of LMR starch samples treated with SS. CK: untreatment LMR, 120−2: LMR treated with SS at 120 °C for 2 min, with comparable conditions applied to other cases.

The utility of X-ray diffraction becomes evident in its ability to identify the densely packed helical structures, denoting crystalline formations within starch granules. Characterized by regular three-dimensional geometrical patterns, these structures enable the assessment of both the relative crystallinity and the long-range structure of starch [38]. Figure 4A and Table 2 present the X-ray diffractograms and relative crystallinity data, respectively, for starch samples derived from SS-processed LMR. The untreated samples exhibited the conventional A-type crystalline pattern observed in starches, featuring two broad single peaks at 15° and 23° (2θ) and dual peaks at 17° and 18° (2θ) [39]. Remarkably, SS treatment induced no significant alterations in the diffraction pattern, suggesting that the crystal form of LMR starch remained unchanged. In contrast to untreated LMR starch, the distinct peak proximate to 20° in SS-treated LMR starch progressively increased in prominence with elevated SS temperature and prolonged treatment duration (Figure 4A). This distinctive pattern represents a classic V-type diffraction, indicative of potential interactions between starch molecules and polar organic substances during the heat treatment process. This suggests the formation of complexes such as amylose–lipid complexes [40].

Prior investigations have reported that the moisture content of rice grains, constrained within the range of 11.1% to 13.8% during hot air processing, prevents starch gelatinization even at elevated temperatures [29,41]. Nonetheless, a noteworthy decline of 5.52% in relative crystallinity was observed in the 160 °C-8 min SS-treated LMR starch. This reduction reflects a disruption in crystalline structure attributable to partial gelatinization [16]. During the early phases of SS processing, an elevation in moisture content is anticipated due to the condensation of SS vapor on the rice grain surface [29]. This likely induces partial gelatinization in SS–LMR, facilitated by the conjunction of initial condensation and elevated temperatures. Subsequent to this initial phase, a continual reduction in LMR moisture content renders starch gelatinization no longer feasible under the evolving conditions.

#### 3.4.2. Short-Range Ordered Structure of Starch

Fourier transform infrared spectroscopy (FTIR) has proven instrumental in characterizing the extent of short-range ordered structures in starch, as evidenced by recent work [42]. The deconvoluted FTIR spectra of the LMR starches, depicted in Figure 4B, reveal specific absorbances at 1047 cm^−1^ and 995 cm^−1^ that correlate with the ordered structure and crystalline region of starch, while the absorption peak at 1022 cm^−1^ is indicative of the disordered and amorphous region of starch. Consequently, the ratios of 1047/1022 cm^−1^ and 995/1022 cm^−1^ are commonly employed to assess alterations in the degree of short-range molecular order and the double helix, respectively [43]. The results presented in Table 2 indicate a decrease in the degree of short-range molecular order with increasing SS temperature and treatment duration. This decline might be attributed to the partial gelatinization of starch induced by elevated temperatures during SS treatment, leading to a decrease in crystallinity and molecular order. A similar trend has been documented in prior SS treatments [44].

Conversely, the degree of double helix content, denoted by the 995/1022 cm^−1^ ratio, experienced an elevation subsequent to SS treatment. This occurrence is likely attributable to the realignment of amylopectin side chains as the starch undergoes heating beyond its glass transition temperature, resulting in the formation of a new ordered double helix structure within amorphous regions [45]. It is crucial to highlight the consistently higher ratio of 995/1022 cm^−1^ compared to 1047/1022 cm^−1^ across all LMR starch samples, providing evidence that not all double helices contribute to crystal formation. The converse trends in the values of 1047/1022 cm^−1^ and 995/1022 cm^−1^ are likely a consequence of the disruption of hydrogen bonds connecting adjacent helices induced by SS treatment, rather than the dissociation of the double helix structure. This inference was supported by analogous observations outlined by Xu et al. [46].

### 3.5. Impact of SS Treatment on Volatile Profiles

**Figure 5:**
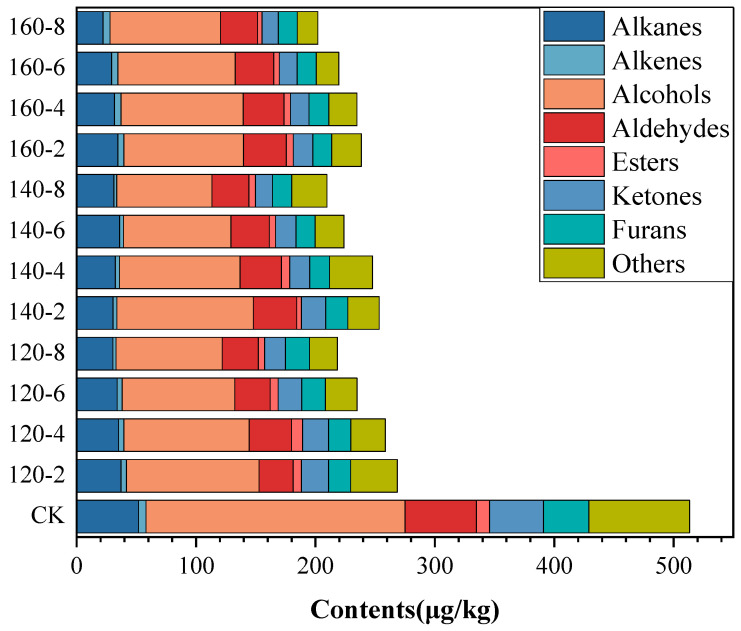
Contents of volatile compounds in LMR treated by SS under different conditions.

The volatile organic compounds extracted from both untreated and SS-treated LMR grains were subjected to analysis using HS-SPME-GC-MS. A total of 59 volatile compounds were identified based on mass spectra data from the NIST 17 library and the Kovats retention index (RI). These compounds encompassed 4 alkanes, 2 alkenes, 10 aldehydes, 14 alcohols, 12 ketones, 5 esters, 5 furans, and 7 compounds categorized under others (Tables S1–S3). Following SS treatment, there was a discernible reduction in the content of each chemical class of compounds in LMR, exhibiting variable degrees of decrease (Figure 5). The reduction in content can be attributed to the ruptured cellular coat, facilitating the rapid release of volatile components under high-temperature conditions [47]. Furthermore, the reduced enzymatic activity observed in SS-treated LMR could potentially decelerate biochemical reactions and/or oxidative degradation processes, consequently resulting in decreased concentrations of volatile compounds.

Alcohols constituted the predominant class of volatiles in untreated LMR, representing 34% of the total volatile compounds. Notably, alcohols such as hexanol, 1-octen-3-ol, and nonanol, characterized by low odor thresholds, significantly contributed to the overall flavor profile of rice [48]. Aldehydes, primarily resulting from lipid oxidation and decomposition, played a pivotal role in the overall flavor profile [49]. Polyunsaturated fatty acids, due to their heightened susceptibility to oxidation, yield higher concentrations of hexanal (21.78 μg/kg) compared to nonanal (13.83 μg/kg) (Tables S1–S3), the latter primarily originating from the oxidation of oleic acid. These outcomes substantiate analogous findings reported in the literature [7,48]. Ketones, with lower odor thresholds, typically impart undesirable flavors to food [50]. The most abundant ketone compound, 6-Methyl-5-hepten-2-one, exhibited a considerable reduction in content (44.7~65.7%) following SS treatment, signifying a decrease in undesirable flavors (Tables S1–S3).

**Figure 6:**
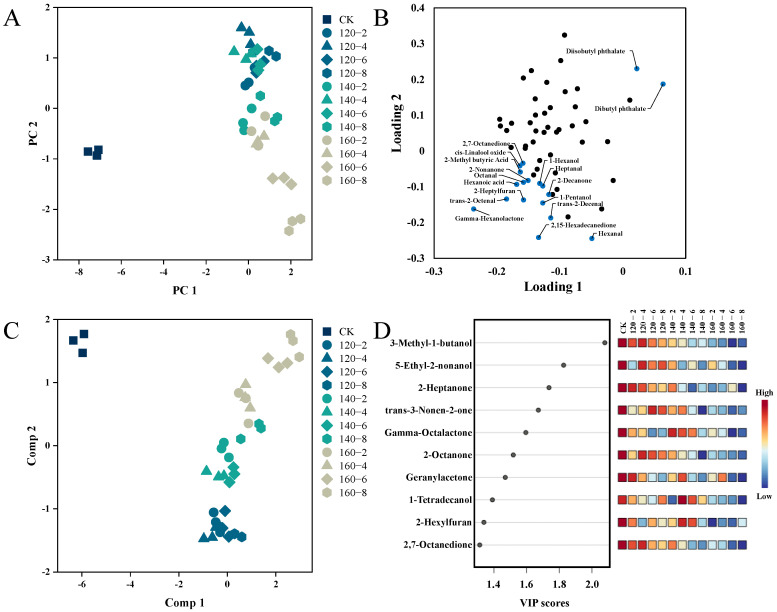
PCA score plot (**A**), PCA loadings plots (**B**), PLS-DA score plot (**C**), and VIP scores (**D**) of the volatile profiles of the LMR. CK: untreatment LMR, 120−2: LMR treated with SS at 120 °C for 2 min, with comparable conditions applied to other cases.

Utilizing Principal Components Analysis (PCA), distinctions among LMR samples exposed to diverse conditions of SS treatment were visually elucidated. Figure 6A demonstrates that the two principal components collectively explain 65.7% of the total variance. A distinct separation is clearly observed between untreated (left) and SS-treated LMR samples (right), accompanied by noticeable shifts among sample groups subjected to distinct SS treatment conditions. Moreover, to further unravel the metabolic differences among these groups, Partial Least Squares Discriminant Analysis (PLS-DA) was employed. The PLS-DA score plot in Figure 6C demonstrates significant inter-group separations, with PC1 and PC2 collectively elucidating 65.1% of the total variance. Additionally, the Variable Importance for the Projection (VIP) was utilized to elucidate the role of each compound in the classification and discrimination of LMR groups. The top 10 discriminants are depicted in Figure 6D, encompassing 5 Ketones (2-Heptanone, 2-Octanone, 2,7-Octanedione, Geranylacetone, trans-3-Nonen-2-one), 3 Alcohols (5-Ethyl-2-nonanol, 3-Methyl-1-butanol, 1-Tetradecanol), 1 Ester (Gamma-Octalactone), and 1 Furan (2-Hexylfuran).

**Figure 7:**
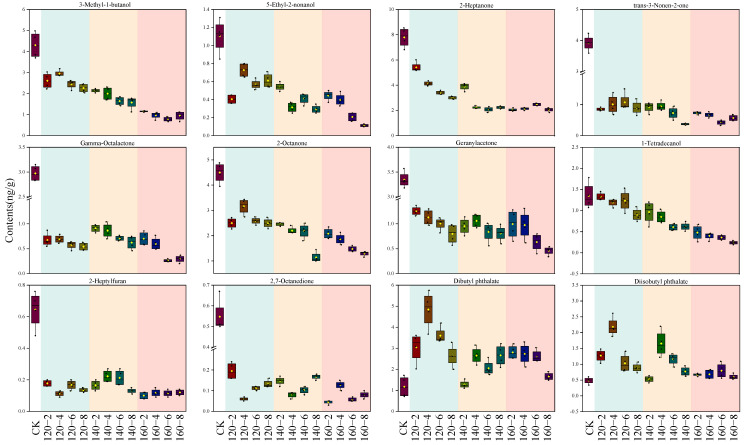
Box plots of 12 characteristic compounds after SS treatment. CK: untreatment LMR, 120−2: LMR treated with SS at 120 °C for 2 min, with comparable conditions applied to other cases.

By integrating results from both the PCA loading plot and the PLS-DA VIP score plot, a total of 12 distinctive compounds were identified. This compilation comprises the top 10 VIP scores and an additional 2 compounds strategically positioned in the upper right quadrant of the PCA loading plot. The alterations in their contents are visually represented using box plots (Figure 7). The first 10 compounds showcased a negative correlation with the temporal extent of SS treatment, contrasting with the final two esters which exhibited a positive correlation with SS treatment time. Esters may originate from reactions involving naturally occurring acids and alcohols. The observed heightened levels of Diisobutyl phthalate and Dibutyl phthalate in this study could be attributed to the facilitated synthesis induced by the SS treatment [49]. The concentrations of 3-Methyl-1-butanol, Tetradecanol, 2-Heptanone, 2-Octanone, and Geranylacetone exhibited a diminishing trend with escalating SS treatment time and temperature. This aligns with the outcomes of a study by Yang et al. [7] on superheated-steam-treated black beans, wherein a marked reduction in the levels of 2-heptanone and 2-octanone was observed. The application of SS in barley cooking, as investigated by Takemitsu et al. [51], yielded analogous outcomes. Utilizing gas chromatography–mass spectrometry for the evaluation of undesirable odors in grains demonstrated substantial mitigation of such odors in SS-based cooking in contrast to conventional cooking methods.

## 4. Conclusions

This investigation has demonstrated the efficacy of SS processing in modulating the water content and distribution in LMR, alongside its effective deactivation of lipase, LOX, and POD activities, thereby facilitating prolonged storage of LMR. Simultaneously, alterations in the color and surface morphology of LMR kernels were observed to varying degrees under different SS treatment conditions. Notably, the application of higher temperatures and/or extended durations of SS exposure resulted in the formation of noticeable fissures. In contrast, only subtle adjustments in crystallinity were observed in response to intensified SS treatment conditions. VOC profile analysis revealed an increase in aromatic esters content and a concurrent reduction in undesirable ketone odors in SS-treated LMR. Taken together, the optimal treatment condition of SS was identified as 120 °C for 6 min in processing LMR, ensuring desired enzyme deactivation and enhanced commercial attributes. Additionally, these findings might contribute to elucidating the relationship between the physicochemical properties and quality characteristics of LMR. Moreover, they provide essential considerations for strategically selecting optimal operational parameters for superheated steam treatment, thereby enhancing the commercial attributes of LMR.

## Figures and Tables

**Table 1:** Effect of SS treatment on color change values (L*—brightness, a*—redness, b*—yellowness).

Treatment Conditions	L*	a*	b*	ΔE*
CK	67.28 ± 0.20 ^e^	2.10 ± 0.11 ^a^	24.47 ± 0.30 ^a^	–
120−2	69.42 ± 0.40 ^d^	2.10 ± 0.15 ^a^	24.30 ± 0.24 ^ab^	2.14 ± 0.19 ^d^
120−4	70.07 ± 0.27 ^cd^	2.17 ± 0.11 ^a^	23.42 ± 0.07 ^c^	2.28 ± 0.20 ^d^
120−6	70.28 ± 0.35 ^cd^	2.20 ± 0.14 ^a^	23.06 ± 0.36 ^cd^	2.32 ± 0.16 ^d^
120−8	71.73 ± 0.48 ^b^	2.20 ± 0.15 ^a^	22.69 ± 0.23 ^de^	2.79 ± 0.28 ^bc^
140−2	70.03 ± 0.14 ^cd^	2.11 ± 0.16 ^a^	23.50 ± 0.27 ^bc^	2.31 ± 0.35 ^d^
140−4	70.16 ± 0.27 ^cd^	2.19 ± 0.13 ^a^	23.28 ± 0.26 ^c^	2.52 ± 0.34 ^d^
140−6	71.22 ± 0.23 ^bc^	2.23 ± 0.16 ^a^	22.82 ± 0.16 ^cd^	2.78 ± 0.28 ^cd^
140−8	71.93 ± 0.21 ^ab^	2.31 ± 0.15 ^a^	22.28 ± 0.21 ^ef^	3.15 ± 0.28 ^ab^
160−2	70.16 ± 0.30 ^cd^	2.12 ± 0.16 ^a^	22.39 ± 0.29 ^de^	2.95 ± 0.49 ^b^
160−4	71.75 ± 0.32 ^b^	2.26 ± 0.13 ^a^	22.07 ± 0.23 ^ef^	4.08 ± 0.54 ^ab^
160−6	72.38 ± 0.33 ^ab^	2.30 ± 0.12 ^a^	22.00 ± 0.21 ^ef^	5.37 ± 0.30 ^ab^
160−8	73.05 ± 0.54 ^a^	2.32 ± 0.10 ^a^	21.83 ± 0.19 ^f^	5.65 ± 0.32 ^a^

Data presented as mean ± standard deviation. For each SS temperature, values with different superscript letters in rows are significantly different (*p* < 0.05). L*—brightness, a*—redness, b*—yellowness. CK: untreatment LMR, 120−2: LMR treated with SS at 120 °C for 2 min, with comparable conditions applied to other cases.

**Table 2:** The ratios of FTIR absorbances at 1047/1022 cm^−1^ and 995/1022 cm^−1^ and relative crystallinity of LMR starch.

Sample	Relative Crystallinity (%)	The Ratio of Absorbances at 1047/1022 cm^−1^	The Ratio of Absorbances at 995/1022 cm^−1^
CK	34.79 ± 0.67 ^a^	0.74 ± 0.01 ^a^	0.73 ± 0.01 ^c^
120−2	33.64 ± 0.26 ^b^	0.74 ± 0.02 ^a^	0.73 ± 0.02 ^c^
120−4	33.36 ± 0.32 ^b^	0.73 ± 0.03 ^ab^	0.73 ± 0.01 ^c^
120−6	33.29 ± 0.44 ^b^	0.73 ± 0.01 ^ab^	0.73 ± 0.02 ^c^
120−8	33.19 ± 0.45 ^b^	0.72 ± 0.01 ^ab^	0.74 ± 0.01 ^bc^
140−2	33.32 ± 0.24 ^b^	0.72 ± 0.02 ^ab^	0.73 ± 0.02 ^c^
140−4	33.10 ± 0.77 ^b^	0.72 ± 0.01 ^ab^	0.75 ± 0.01 ^ab^
140−6	33.05 ± 0.52 ^b^	0.72 ± 0.01 ^ab^	0.74 ± 0.01 ^bc^
140−8	32.98 ± 0.31 ^b^	0.72 ± 0.02 ^ab^	0.74 ± 0.02 ^bc^
160−2	33.08 ± 0.56 ^b^	0.72 ± 0.01 ^ab^	0.74 ± 0.01 ^bc^
160−4	32.92 ± 0.34 ^b^	0.72 ± 0.01 ^ab^	0.76 ± 0.01 ^ab^
160−6	32.88 ± 0.40 ^b^	0.72 ± 0.01 ^ab^	0.75 ± 0.01 ^ab^
160−8	32.87 ± 0.59 ^b^	0.71 ± 0.02 ^b^	0.77 ± 0.01 ^a^

Data presented as mean ± standard deviation. For each SS temperature, values with different superscript letters in rows are significantly different (*p* < 0.05). CK: untreatment LMR, 120−2: LMR treated with SS at 120 °C for 2 min, with comparable conditions applied to other cases.

## Data Availability

Data is contained within the article or Supplementary Materials.

## Supplementary Materials

The following supporting information can be downloaded at: https://www.mdpi.com/article/10.3390/foods13020240/s1, Table S1. List of volatiles (ng/g) identified by HS-SPME-GC/MS for the untreated LMR and SS-treated LMR under 120 °C. Table S2. List of volatiles (ng/g) identified by HS-SPME-GC/MS for the untreated LMR and SS-treated LMR under 140 °C. Table S3. List of volatiles (ng/g) identified by HS-SPME-GC/MS for the untreated LMR and SS-treated LMR under 160 °C.
