# Supplementary material for: A Comprehensive Study on the Influence of Superheated Steam Treatment on Lipolytic Enzymes, Physicochemical Characteristics, and Volatile Composition of Lightly Milled Rice
Source: Foods. 2024 Jan 11;13(2):240. doi: 10.3390/foods13020240 (PMC10815025; doi:10.3390/foods13020240)
Supplement: Supplementary file 1 [file foods-13-00240-s001.zip › foods-2800501-supplementary.pdf]

Supplementary Materials

A Comprehensive Study on the Influence of Superheated Steam Treatment on Lipolytic Enzymes, Physicochemical Characteristics, and Volatile Composition of Lightly Milled Rice

Chenguang Zhou [1], Bin Li [1], Wenli Yang [1], Tianrui Liu [1], Haoran Yu [1] and Siyao Liu [2,*], Zhen Yang [3,*]

[1] Agricultural Product Processing and Storage Lab, School of Food and Biological Engineering, Jiangsu University, Zhenjiang 212013, China
[2] School of Pharmacy, Jiangsu University, Zhenjiang 212013, China
[3] Key Laboratory of Nuclear Agricultural Sciences of Ministry of Agriculture and Zhejiang Province, Institute of Nuclear Agricultural Sciences, Zhejiang University, Hangzhou 310058, China

* Correspondence: siyaoliu@ujs.edu.cn (S.L.); zhen.yang@zju.edu.cn (Z.Y.)

Table S1 List of volatiles (ng/g) identified by HS-SPME-GC/MS for the untreated LMR and SS-treated LMR under 120 °C.

Volatile compounds	RT	RI cal[1]	RI ref[2]	CK	120-2	120-4	120-6	120-8
**Alkanes**								
5-Methylundecane	9.875	1142	1152	9.36±1.40[a]	5.35±0.68[c]	9.32±0.91[ab]	6.67±0.24[c]	7.16±0.63[bc]
Dodecane	11.655	1194		29.83±3.05[a]	16.86±1.88[b]	25.05±2.26[ab]	17.88±0.70[b]	19.70±2.55[b]
Tetradecane	18.02	1393		7.16±0.28[a]	3.84±0.40[c]	6.33±0.57[ab]	5.38±0.82[bc]	4.50±0.65[c]
Pentadecane	20.82	1494		5.55±0.47[a]	4.34±0.72[b]	4.57±0.18[ab]	4.02±0.16[b]	3.79±0.09[b]
**Alkenes**								
D-Limonene	11.245	1182		5.57±0.41[a]	2.43±0.25[c]	4.28±0.27[b]	3.86±0.54[b]	4.25±0.21[b]
cis-5-Tridecene	15.36	1306		0.58±0.10[a]	0.21±0.03[b]	0.24±0.04[b]	0.27±0.03[b]	0.27±0.03[b]
**Alcohols**								
2-Propyl-1-pentanol	6.515	1043		1.60±0.16[a]	0.93±0.10[b]	1.14±0.08[b]	1.04±0.05[b]	1.12±0.08[b]
2-Propyl-1-heptanol	9.005	1116		1.69±0.26[a]	0.83±0.04[b]	0.86±0.09[b]	0.80±0.05[b]	0.79±0.06[b]
5-Ethyl-2-nonanol	9.145	1120		1.10±0.17[a]	0.40±0.04[c]	0.73±0.07[b]	0.57±0.05[bc]	0.61±0.07[b]
3-Methyl-1-butanol	11.895	1201	1202	4.30±0.54[a]	2.61±0.32[b]	2.95±0.13[b]	2.45±0.18[b]	2.27±0.18[b]
2-Hexanol	12.37	1215	1217	0.54±0.07[ab]	0.43±0.02[c]	0.64±0.05[a]	0.52±0.02[bc]	0.58±0.04[bc]
1-Pentanol	13.25	1242	1241	25.14±1.27[a]	11.47±0.86[b]	9.72±0.32[bc]	8.73±0.32[cd]	7.90±0.38[d]
2-Heptanol	15.445	1308	1310	2.43±0.21[a]	1.38±0.06[b]	1.35±0.17[b]	1.10±0.07[bc]	0.93±0.06[c]
1-Hexanol	16.415	1340	1340	137.62±8.23[a]	68.69±4.54[b]	61.58±2.06[bc]	55.23±1.32[bc]	50.80±3.37[c]
1-Octen-3-ol	19.225	1436	1436	7.81±0.24[a]	4.16±0.26[bc]	4.44±0.14[b]	3.77±0.25[c]	3.70±0.29[c]
1-Heptanol	19.34	1440	1440	11.76±0.57[a]	5.52±0.55[b]	5.39±0.32[b]	5.17±0.22[b]	4.81±0.35[b]
6-Methyl-5-hepten-2-ol	19.565	1448	1451	7.13±0.48[a]	4.61±0.52[b]	4.86±0.35[b]	4.65±0.11[b]	4.54±0.17[b]
1-Octanol	22.11	1544	1545	10.33±0.62[a]	5.70±0.48[b]	6.21±0.43[b]	5.94±0.34[b]	6.01±0.55[b]
1-Nonanol	24.72	1648	1649	4.00±0.65[a]	3.30±0.58[a]	4.12±0.22[a]	3.78±0.75[a]	3.89±0.90[a]
1-Tetradecanol	35.975	2152	2152	1.35±0.27[a]	0.96±0.22[ab]	0.85±0.13[ab]	0.61±0.07[b]	0.92±0.02[ab]
**Aldehydes**								

Hexanal	7.525	1072	1072	21.78±1.37[a]	12.07±1.04[b]	11.21±0.30[b]	10.13±0.50[b]	10.49±0.76[b]
Heptanal	10.955	1173	1176	3.30±0.06[a]	1.54±0.10[b]	1.42±0.06[b]	1.24±0.10[bc]	1.08±0.11[c]
2-Hexenal	13.065	1236	1248	4.44±0.36[a]	1.87±0.12[c]	3.08±0.35[b]	2.24±0.05[c]	2.40±0.16[bc]
Octanal	14.34	1274	1275	5.02±0.14[a]	1.80±0.18[b]	1.78±0.35[b]	1.35±0.09[b]	1.36±0.07[b]
trans-2-Heptenal	15.295	1303	1306	2.31±0.07[a]	0.77±0.11[bc]	0.92±0.10[b]	0.59±0.11[c]	0.58±0.07[c]
Nonanal	17.525	1377	1379	13.83±0.21[a]	7.92±0.41[b]	13.58±0.95[a]	11.16±1.21[a]	11.47±0.60[a]
trans-2-Octenal	18.445	1408	1412	4.28±0.52[a]	0.71±0.10[b]	0.68±0.11[b]	0.46±0.05[b]	0.55±0.10[b]
Decanal	20.515	1483	1483	1.69±0.08[a]	0.93±0.12[c]	1.70±0.36[ab]	1.27±0.19[bc]	1.01±0.12[bc]
trans-2-Decenal	24.21	1627	1629	2.66±0.16[a]	0.77±0.10[b]	0.84±0.06[b]	0.83±0.05[b]	0.80±0.04[b]
2-Undecenal	26.855	1738	1740	0.52±0.06[a]	0.17±0.04[d]	0.31±0.03[c]	0.34±0.02[bc]	0.44±0.06[ab]
**Esters**								
Gamma-Hexanolactone	25.41	1677	1678	2.58±0.25[a]	0.37±0.05[b]	0.11±0.02[b]	0.13±0.01[b]	0.04±0.01[b]
Gamma-Octalactone	30.385	1891	1889	2.97±0.14[a]	0.67±0.12[b]	0.69±0.07[b]	0.58±0.07[b]	0.54±0.07[b]
Gamma-Nonanolactone	32.81	2000	2003	3.86±0.11[a]	1.62±0.16[b]	1.38±0.02[bc]	1.36±0.18[cd]	1.24±0.09[d]
Diisobutyl phthalate	42.575	2470	2526	0.49±0.09[c]	1.27±0.16[b]	2.18±0.27[a]	1.03±0.25[bc]	0.89±0.12[bc]
Dibutyl phthalate	44.995	2586	2592	1.17±0.43[c]	3.05±0.62[bc]	4.84±0.76[a]	3.60±0.35[ab]	2.63±0.45[bc]
**Ketones**								
2-Heptanone	10.865	1171	1172	7.80±0.68[a]	5.41±0.35[b]	4.14±0.15[c]	3.41±0.13[cd]	3.00±0.10[d]
6-Methyl-2-heptanone	12.71	1225	1228	1.66±0.10[a]	0.91±0.13[b]	0.77±0.02[b]	0.70±0.02[b]	0.67±0.05[b]
2-Octanone	14.21	1270	1275	4.50±0.36[a]	2.49±0.17[c]	3.16±0.27[b]	2.58±0.12[bc]	2.49±0.16[bc]
1-Decen-3-one	14.71	1286		0.53±0.06[a]	0.29±0.03[b]	0.41±0.08[b]	0.23±0.04[b]	0.36±0.06[b]
2,5-Octanedione	15.52	1311	1325	0.16±0.03[b]	0.31±0.02[a]	0.25±0.03[a]	0.17±0.02[b]	0.19±0.03[b]
6-Methyl-5-hepten-2-one	15.825	1321	1323	17.68±1.55[a]	9.77±0.53[b]	10.65±0.51[b]	9.25±0.26[b]	9.07±0.56[b]
2,7-Octanedione	15.94	1325		0.55±0.07[a]	0.19±0.03[b]	0.06±0.01[c]	0.11±0.01[bc]	0.13±0.01[bc]
2-Nonanone	17.385	1372	1374	1.32±0.08[a]	0.48±0.08[b]	0.48±0.06[b]	0.47±0.09[b]	0.41±0.06[b]
2,15-Hexadecanedione	18.315	1403		2.62±0.48[a]	0.35±0.02[b]	0.34±0.05[b]	0.29±0.03[b]	0.35±0.06[b]

2-Decanone	20.365	1477	1482	0.99±0.12[a]	0.42±0.07[b]	0.29±0.06[b]	0.33±0.08[b]	0.26±0.04[b]
Geranylacetone	29.275	1842	1843	3.34±0.14[a]	1.26±0.08[b]	1.13±0.13[bc]	1.01±0.11[bc]	0.80±0.17[c]
trans-3-Nonen-2-one	33.77	2046		3.92±0.23[a]	0.85±0.04[b]	1.01±0.26[b]	1.08±0.25[b]	0.90±0.19[b]
**Furans**								
2-Pentylfuran	12.57	1221	1222	26.18±2.74[a]	14.29±1.50[b]	14.58±1.10[b]	15.61±0.55[b]	16.19±0.77[b]
2-Hexylfuran	15.725	1318	1323	0.65±0.11[a]	0.18±0.02[b]	0.12±0.01[b]	0.17±0.03[b]	0.13±0.01[b]
2-Heptylfuran	18.76	1419	1429	0.79±0.04[a]	0.16±0.03[bc]	0.11±0.02[c]	0.17±0.02[bc]	0.23±0.03[b]
cis-Linalool oxide	18.91	1425	1425	6.43±0.32[a]	2.05±0.17[b]	2.21±0.13[b]	2.11±0.12[b]	2.01±0.24[b]
trans-Linalool oxide	19.695	1453	1452	4.09±0.23[a]	1.72±0.22[b]	1.66±0.20[b]	1.73±0.15[b]	1.53±0.08[b]
**Others**								
Toluene	6.2	1033	1033	7.12±0.78[b]	16.07±1.95[a]	9.23±0.35[b]	8.67±0.50[b]	7.78±0.42[b]
1,3-dimethylbenzene	10.73	1167	1164	1.33±0.08[a]	1.15±0.16[a]	1.31±0.08[a]	1.33±0.06[a]	1.22±0.03[a]
N,N-Dimethylformamide	15.23	1301		0.26±0.03[a]	0.09±0.02[b]	0.10±0.02[b]	0.10±0.01[b]	0.08±0.01[b]
2-Methyl butyric Acid	24.765	1650	1652	4.60±0.59[a]	1.16±0.19[b]	1.41±0.24[b]	1.44±0.18[b]	0.94±0.07[b]
Pentanoic acid	26.385	1718	1719	6.13±0.84[a]	3.38±0.53[b]	3.30±0.25[b]	3.08±0.38[b]	3.02±0.07[b]
Hexanoic acid	28.88	1825	1825	52.66±1.70[a]	15.71±3.73[b]	12.03±0.86[b]	11.23±1.36[b]	9.19±0.67[b]
Nonanoic acid	35.665	2137	2137	12.17±2.52[a]	1.53±0.30[b]	1.53±0.19[b]	0.75±0.10[b]	1.17±0.21[b]

[1] RI_{cal}, the experimental Kovat's retention index calculated based on a DB-WAX capillary column.

[2] RI_{ref}, the Kovats' retention index information obtained from the NIST Chemistry WebBook database (https://webbook.nist.gov/chemistry/name-ser/).

Data were presented as mean ± standard deviation. For each SS treatment time, values with different superscript letters in rows were significantly different (p<0.05).

Table S2. List of volatiles (ng/g) identified by HS-SPME-GC/MS for the untreated LMR and SS-treated LMR under 140 °C.

Volatile compounds	RT	RI $_{cal}$[1]	RI $_{ref}$[2]	CK	140-2	140-4	140-6	140-8
**Alkanes**								
5-Methylundecane	9.875	1142	1152	9.36±1.40^{a}	5.89±0.27^{b}	5.26±0.23^{b}	5.72±0.58^{b}	3.28±0.26^{c}
Dodecane	11.655	1194		29.83±3.05^{a}	16.12±1.47^{b}	15.34±1.85^{b}	19.12±2.20^{b}	16.97±0.70^{b}
Tetradecane	18.02	1393		7.16±0.28^{a}	5.13±0.74^{b}	7.08±0.75^{ab}	6.90±0.41^{ab}	6.05±0.30^{ab}
Pentadecane	20.82	1494		5.55±0.47^{a}	3.34±0.34^{b}	4.66±0.18^{ab}	4.18±0.30^{ab}	4.88±0.90^{ab}
**Alkenes**								
D-Limonene	11.245	1182		5.57±0.41^{a}	3.04±0.14^{b}	3.33±0.32^{b}	3.08±0.04^{b}	3.10±0.08^{b}
cis-5-Tridecene	15.36	1306		0.58±0.10^{a}	0.23±0.02^{b}	0.16±0.02^{b}	0.24±0.04^{b}	0.20±0.03^{b}
**Alcohols**								
2-Propyl-1-pentanol	6.515	1043		1.60±0.16^{a}	1.08±0.03^{b}	1.04±0.08^{b}	1.04±0.08^{b}	0.90±0.03^{b}
2-Propyl-1-heptanol	9.005	1116		1.69±0.26^{a}	0.71±0.04^{b}	0.74±0.05^{b}	0.70±0.04^{b}	0.71±0.07^{b}
5-Ethyl-2-nonanol	9.145	1120		1.10±0.17^{a}	0.54±0.04^{b}	0.32±0.05^{c}	0.41±0.05^{bc}	0.29±0.04^{c}
3-Methyl-1-butanol	11.895	1201	1202	4.30±0.54^{a}	2.14±0.07^{b}	2.00±0.27^{b}	1.65±0.17^{b}	1.57±0.26^{b}
2-Hexanol	12.37	1215	1217	0.54±0.07^{a}	0.53±0.07^{a}	0.38±0.04^{ab}	0.46±0.07^{ab}	0.28±0.04^{b}
Pentanol	13.25	1242	1241	25.14±1.27^{a}	12.27±0.86^{b}	10.20±0.41^{bc}	9.02±0.16^{c}	9.98±1.21^{bc}
2-Heptanol	15.445	1308	1310	2.43±0.21^{a}	1.42±0.14^{b}	1.07±0.14^{bc}	0.94±0.09^{c}	0.88±0.04^{c}
Hexanol	16.415	1340	1340	137.62±8.23^{a}	70.81±3.55^{b}	60.07±2.78^{bc}	53.28±1.47^{cd}	44.98±1.86^{d}
1-Octen-3-ol	19.225	1436	1436	7.81±0.24^{a}	4.92±0.10^{b}	4.55±0.26^{bc}	4.08±0.06^{c}	4.06±0.19^{c}
Heptanol	19.34	1440	1440	11.76±0.57^{a}	5.64±0.18^{b}	5.44±0.24^{b}	4.82±0.17^{bc}	3.75±0.53^{c}
6-Methyl-5-hepten-2-ol	19.565	1448	1451	7.13±0.48^{a}	4.66±0.14^{b}	4.54±0.22^{bc}	3.83±0.07^{cd}	3.67±0.12^{d}
1-Octanol	22.11	1544	1545	10.33±0.62^{a}	5.90±0.26^{b}	5.94±0.53^{b}	5.14±0.47^{b}	4.53±0.99^{b}
1-Nonanol	24.72	1648	1649	4.00±0.65^{a}	3.32±0.35^{a}	3.50±0.28^{a}	3.34±0.25^{a}	3.24±0.14^{a}
1-Tetradecanol	35.975	2152	2152	1.35±0.27^{a}	0.25±0.01^{b}	1.19±0.08^{a}	1.23±0.21^{a}	0.90±0.13^{a}
**Aldehydes**								

Hexanal	7.525	1072	1072	21.78±1.37^{a}	12.89±0.46^{c}	11.08±0.72^{cd}	10.33±0.29^{d}	15.49±2.15^{b}
Heptanal	10.955	1173	1176	3.30±0.06^{a}	1.49±0.21^{bc}	1.32±0.10^{bc}	1.19±0.07^{c}	1.67±0.22^{b}
2-Hexenal	13.065	1236	1248	4.44±0.36^{a}	2.13±0.14^{b}	2.03±0.21^{b}	2.10±0.22^{b}	1.62±0.17^{b}
Octanal	14.34	1274	1275	5.02±0.14^{a}	1.65±0.04^{bc}	1.80±0.13^{b}	1.42±0.09^{bc}	1.40±0.02^{c}
trans-2-Heptenal	15.295	1303	1306	2.31±0.07^{a}	0.95±0.05^{b}	0.62±0.09^{cd}	0.77±0.14^{bc}	0.39±0.08^{d}
Nonanal	17.525	1377	1379	13.83±0.21^{ab}	14.33±0.56^{a}	14.40±1.08^{ab}	12.89±0.50^{ab}	12.17±0.18^{b}
trans-2-Octenal	18.445	1408	1412	4.28±0.52^{a}	0.68±0.07^{b}	0.78±0.12^{b}	0.73±0.11^{b}	0.80±0.12^{b}
Decanal	20.515	1483	1483	1.69±0.08^{a}	1.17±0.19^{ab}	1.36±0.14^{ab}	1.47±0.17^{a}	0.98±0.16^{b}
trans-2-Decenal	24.21	1627	1629	2.66±0.16^{a}	0.75±0.03^{b}	0.92±0.07^{b}	0.82±0.13^{b}	1.06±0.12^{b}
2-Undecenal	26.855	1738	1740	0.52±0.06^{a}	0.33±0.06^{b}	0.36±0.05^{ab}	0.41±0.04^{ab}	0.27±0.04^{b}
**Esters**								
Gamma-Hexanolactone	25.41	1677	1678	2.58±0.25^{a}	0.14±0.04^{b}	0.22±0.05^{b}	0.12±0.02^{b}	0.10±0.01^{b}
Gamma-Octalactone	30.385	1891	1889	2.97±0.14^{a}	0.91±0.07^{b}	0.86±0.13^{bc}	0.71±0.05^{bc}	0.63±0.12^{c}
Gamma-Nonanolactone	32.81	2000	2003	3.86±0.11^{a}	1.45±0.26^{b}	1.55±0.11^{b}	1.23±0.17^{b}	1.38±0.17^{b}
Diisobutyl phthalate	42.575	2470	2526	0.49±0.09^{bc}	0.13±0.03^{c}	1.65±0.36^{a}	1.16±0.16^{a}	0.77±0.12^{b}
Dibutyl phthalate	44.995	2586	2592	1.17±0.43^{b}	1.27±0.16^{b}	2.65±0.34^{a}	2.05±0.30^{ab}	2.66±0.44^{a}
**Ketones**								
2-Heptanone	10.865	1171	1172	7.80±0.68^{a}	3.89±0.25^{b}	2.23±0.09^{c}	2.09±0.17^{c}	2.23±0.09^{c}
6-Methyl-2-heptanone	12.71	1225	1228	1.66±0.10^{a}	0.85±0.07^{b}	0.60±0.03^{b}	0.59±0.10^{b}	0.68±0.13^{b}
2-Octanone	14.21	1270	1275	4.50±0.36^{a}	2.45±0.06^{b}	2.19±0.12^{b}	2.18±0.25^{b}	1.14±0.18^{c}
1-Decen-3-one	14.71	1286		0.53±0.06^{a}	0.30±0.05^{bc}	0.41±0.04^{ab}	0.35±0.08^{bc}	0.20±0.04^{c}
2,5-Octanedione	15.52	1311	1325	0.16±0.03^{b}	0.27±0.02^{a}	0.26±0.04^{a}	0.24±0.04^{ab}	0.21±0.04^{ab}
6-Methyl-5-hepten-2-one	15.825	1321	1323	17.68±1.55^{a}	9.40±0.10^{b}	7.75±0.40^{bc}	7.44±0.37^{c}	7.33±0.76^{c}
2,7-Octanedione	15.94	1325		0.55±0.07^{a}	0.15±0.02^{b}	0.08±0.01^{b}	0.10±0.02^{b}	0.17±0.01^{b}
2-Nonanone	17.385	1372	1374	1.32±0.08^{a}	0.56±0.03^{b}	0.50±0.05^{b}	0.43±0.07^{b}	0.40±0.01^{b}
2,15-Hexadecanedione	18.315	1403		2.62±0.48^{a}	0.39±0.07^{b}	0.38±0.04^{b}	0.81±0.07^{b}	0.34±0.06^{b}

2-Decanone	20.365	1477	1482	0.99±0.12[a]	0.26±0.05[c]	0.47±0.04[b]	0.24±0.06[c]	0.40±0.04[bc]
Geranylacetone	29.275	1842	1843	3.34±0.14[a]	0.95±0.14[b]	1.06±0.12[b]	0.84±0.17[b]	0.81±0.14[b]
trans-3-Nonen-2-one	33.77	2046		3.92±0.23[a]	0.92±0.15[b]	0.95±0.13[b]	0.73±0.16[b]	0.37±0.02[c]
**Furans**								
2-Pentylfuran	12.57	1221	1222	26.18±2.74[a]	13.95±0.37[b]	11.84±0.63[b]	12.30±0.76[b]	13.22±0.43[b]
2-Hexylfuran	15.725	1318	1323	0.65±0.11[a]	0.16±0.03[bc]	0.22±0.03[b]	0.21±0.04[bc]	0.13±0.01[c]
2-Heptylfuran	18.76	1419	1429	0.79±0.04[a]	0.14±0.01[bc]	0.18±0.04[b]	0.07±0.01[c]	0.12±0.02[bc]
cis-Linalool oxide	18.91	1425	1425	6.43±0.32[a]	2.41±0.12[b]	2.37±0.18[bc]	1.92±0.11[cd]	1.51±0.17[d]
trans-Linalool oxide	19.695	1453	1452	4.09±0.23[a]	1.73±0.09[b]	1.88±0.09[b]	1.57±0.08[b]	1.19±0.13[c]
**Others**								
Toluene	6.2	1033	1033	7.12±0.78[a]	5.41±0.13[b]	4.42±0.32[bc]	4.28±0.33[b]	2.83±0.42[c]
1,3-dimethylbenzene	10.73	1167	1164	1.33±0.08[a]	1.25±0.09[a]	1.33±0.09[a]	1.40±0.08[a]	1.23±0.03[a]
N,N-Dimethylformamide	15.23	1301		0.26±0.03[a]	0.08±0.01[c]	0.08±0.01[c]	0.10±0.01[c]	0.17±0.02[b]
2-Methyl butyric Acid	24.765	1650	1652	4.60±0.59[a]	1.21±0.12[b]	1.48±0.16[b]	1.53±0.05[b]	1.23±0.17[b]
Pentanoic acid	26.385	1718	1719	6.13±0.84[a]	2.79±0.25[c]	3.63±0.12[b]	2.89±0.31[bc]	3.21±0.25[bc]
Hexanoic acid	28.88	1825	1825	52.66±1.70[a]	14.51±0.80[b]	16.06±1.19[b]	11.68±1.24[b]	15.15±1.62[b]
Nonanoic acid	35.665	2137	2137	12.17±2.52[a]	1.03±0.19[d]	9.00±0.61[b]	2.38±0.61[d]	5.65±0.67[c]

[1] RI_{cal}, the experimental Kovat's retention index calculated based on a DB-WAX capillary column.

[2] RI_{ref}, the Kovats' retention index information obtained from the NIST Chemistry WebBook database (https://webbook.nist.gov/chemistry/name-ser/).

Data were presented as mean ± standard deviation. For each SS treatment time, values with different superscript letters in rows were significantly different (p<0.05).

Table S3. List of volatiles (ng/g) identified by HS-SPME-GC/MS for the untreated LMR and SS-treated LMR under 160 °C.

Volatile compounds	RT	RI $_{cal}$ [1]	RI $_{ref}$ [2]	CK	160-2	160-4	160-6	160-8
**Alkanes**								
5-Methylundecane	9.875	1142	1152	9.36±1.40^{a}	5.43±0.39^{b}	4.61±0.77^{bc}	3.88±0.34^{cd}	2.75±0.29^{d}
Dodecane	11.655	1194		29.83±3.05^{a}	18.84±1.51^{b}	17.78±2.37^{b}	16.97±1.62^{b}	12.82±0.68^{b}
Tetradecane	18.02	1393		7.16±0.28^{a}	6.22±0.61^{ab}	5.72±0.77^{ab}	5.29±0.18^{bc}	3.87±0.24^{c}
Pentadecane	20.82	1494		5.55±0.47^{a}	4.05±0.37^{b}	3.61±0.23^{b}	3.25±0.50^{b}	2.68±0.56^{b}
**Alkenes**								
D-Limonene	11.245	1182		5.57±0.41^{a}	4.75±0.62^{a}	5.12±0.30^{a}	4.71±0.40^{a}	5.63±0.60^{a}
cis-5-Tridecene	15.36	1306		0.58±0.10^{a}	0.33±0.05^{b}	0.26±0.07^{b}	0.37±0.05^{b}	0.21±0.04^{b}
**Alcohols**								
2-Propyl-1-pentanol	6.515	1043		1.60±0.16^{a}	0.86±0.02^{b}	0.80±0.11^{b}	0.75±0.05^{b}	0.87±0.06^{b}
2-Propyl-1-heptanol	9.005	1116		1.69±0.26^{a}	0.76±0.09^{b}	0.75±0.12^{b}	0.67±0.09^{b}	0.76±0.06^{b}
5-Ethyl-2-nonanol	9.145	1120		1.10±0.17^{a}	0.45±0.05^{b}	0.40±0.06^{bc}	0.21±0.04^{cd}	0.11±0.02^{d}
3-Methyl-1-butanol	11.895	1201	1202	4.30±0.54^{a}	1.16±0.03^{b}	0.97±0.14^{b}	0.78±0.09^{b}	0.95±0.18^{b}
2-Hexanol	12.37	1215	1217	0.54±0.07^{a}	0.47±0.01^{ab}	0.35±0.05^{bc}	0.35±0.02^{c}	0.27±0.03^{c}
Pentanol	13.25	1242	1241	25.14±1.27^{a}	10.44±0.86^{b}	11.11±0.33^{b}	11.54±0.74^{b}	10.99±0.99^{b}
2-Heptanol	15.445	1308	1310	2.43±0.21^{a}	1.02±0.07^{b}	0.95±0.07^{b}	0.97±0.06^{b}	0.81±0.12^{b}
Hexanol	16.415	1340	1340	137.62±8.23^{a}	60.79±4.10^{b}	62.81±1.62^{b}	63.84±5.36^{b}	59.04±4.24^{b}
1-Octen-3-ol	19.225	1436	1436	7.81±0.24^{a}	5.54±0.32^{b}	5.97±0.19^{b}	4.06±0.18^{c}	3.18±0.27^{d}
Heptanol	19.34	1440	1440	11.76±0.57^{a}	5.17±0.27^{b}	5.27±0.12^{b}	5.08±0.37^{b}	4.68±0.35^{b}
6-Methyl-5-hepten-2-ol	19.565	1448	1451	7.13±0.48^{a}	4.25±0.20^{b}	4.08±0.20^{bc}	3.44±0.21^{cd}	3.06±0.13^{d}
1-Octanol	22.11	1544	1545	10.33±0.62^{a}	5.48±0.59^{bc}	5.30±0.39^{b}	3.95±0.17^{c}	4.41±0.39^{bc}
1-Nonanol	24.72	1648	1649	4.00±0.65^{a}	3.26±0.19^{ab}	3.16±0.30^{ab}	2.28±0.31^{b}	3.18±0.30^{ab}
1-Tetradecanol	35.975	2152	2152	1.35±0.27^{a}	0.48±0.15^{b}	0.40±0.07^{b}	0.36±0.05^{b}	0.24±0.04^{b}
**Aldehydes**								

Hexanal	7.525	1072	1072	21.78±1.37[a]	12.91±1.32[c]	16.11±0.31[bc]	19.81±1.45[ab]	17.63±1.20[b]
Heptanal	10.955	1173	1176	3.30±0.06[a]	1.49±0.08[b]	1.53±0.14[b]	1.33±0.04[b]	1.28±0.07[b]
2-Hexenal	13.065	1236	1248	4.44±0.36[a]	2.15±0.31[b]	1.88±0.23[b]	1.72±0.16[bc]	1.19±0.10[c]
Octanal	14.34	1274	1275	5.02±0.14[a]	1.70±0.15[b]	1.81±0.21[b]	1.37±0.07[b]	1.48±0.10[b]
trans-2-Heptenal	15.295	1303	1306	2.31±0.07[a]	1.13±0.12[c]	1.34±0.12[b]	0.91±0.08[c]	0.39±0.05[d]
Nonanal	17.525	1377	1379	13.83±0.21[a]	13.88±1.25[a]	13.49±0.96[a]	13.04±1.08[a]	12.95±0.94[a]
trans-2-Octenal	18.445	1408	1412	4.28±0.52[a]	0.48±0.10[b]	0.73±0.06[b]	0.78±0.16[b]	0.73±0.07[b]
Decanal	20.515	1483	1483	1.69±0.08[a]	1.46±0.32[ab]	1.18±0.13[ab]	1.01±0.21[b]	1.00±0.10[b]
trans-2-Decenal	24.21	1627	1629	2.66±0.16[a]	1.47±0.40[b]	0.90±0.12[b]	0.99±0.09[b]	1.31±0.17[b]
2-Undecenal	26.855	1738	1740	0.52±0.06[a]	0.09±0.01[c]	0.24±0.04[b]	0.28±0.06[b]	0.10±0.03[c]
**Esters**								
Gamma-Hexanolactone	25.41	1677	1678	2.58±0.25[a]	0.21±0.04[b]	0.17±0.02[b]	0.11±0.02[b]	0.13±0.00[b]
Gamma-Octalactone	30.385	1891	1889	2.97±0.14[a]	0.70±0.12[b]	0.60±0.11[b]	0.26±0.03[c]	0.29±0.06[c]
Gamma-Nonanolactone	32.81	2000	2003	3.86±0.11[a]	1.48±0.25[b]	1.25±0.22[bc]	0.91±0.06[c]	0.96±0.15[c]
Diisobutyl phthalate	42.575	2470	2526	0.49±0.09[a]	0.67±0.04[a]	0.68±0.13[a]	0.79±0.21[a]	0.60±0.07[a]
Dibutyl phthalate	44.995	2586	2592	1.17±0.43[b]	2.80±0.29[a]	2.73±0.43[a]	2.62±0.26[a]	1.66±0.16[b]
**Ketones**								
2-Heptanone	10.865	1171	1172	7.80±0.68[a]	2.05±0.10[b]	2.14±0.09[b]	2.47±0.10[b]	2.05±0.14[b]
6-Methyl-2-heptanone	12.71	1225	1228	1.66±0.10[a]	0.67±0.11[b]	0.73±0.11[b]	0.85±0.08[b]	0.84±0.08[b]
2-Octanone	14.21	1270	1275	4.50±0.36[a]	2.08±0.18[b]	1.86±0.18[b]	1.47±0.09[c]	1.29±0.08[c]
1-Decen-3-one	14.71	1286		0.53±0.06[a]	0.30±0.06[b]	0.17±0.02[b]	0.17±0.01[b]	0.25±0.04[b]
2,5-Octanedione	15.52	1311	1325	0.16±0.03[a]	0.24±0.03[a]	0.26±0.02[a]	0.19±0.03[a]	0.19±0.09[a]
6-Methyl-5-hepten-2-one	15.825	1321	1323	17.68±1.55[a]	7.64±0.47[b]	7.41±0.34[bc]	7.10±0.33[bc]	6.07±0.25[c]
2,7-Octanedione	15.94	1325		0.55±0.07[a]	0.04±0.01[b]	0.13±0.02[b]	0.06±0.01[b]	0.08±0.01[b]
2-Nonanone	17.385	1372	1374	1.32±0.08[a]	0.49±0.03[b]	0.50±0.06[b]	0.45±0.03[b]	0.40±0.06[b]
2,15-Hexadecanedione	18.315	1403		2.62±0.48[a]	0.71±0.08[b]	0.47±0.10[b]	0.85±0.17[b]	0.81±0.11[b]

2-Decanone	20.365	1477	1482	0.99±0.12[a]	0.48±0.06[c]	0.20±0.04[d]	0.23±0.03[d]	0.64±0.10[b]
Geranylacetone	29.275	1842	1843	3.34±0.14[a]	0.99±0.25[b]	0.98±0.25[b]	0.64±0.15[bc]	0.46±0.07[c]
trans-3-Nonen-2-one	33.77	2046		3.92±0.23[a]	0.74±0.04[b]	0.67±0.07[b]	0.42±0.06[b]	0.57±0.08[b]
**Furans**								
2-Pentylfuran	12.57	1221	1222	26.18±2.74[a]	11.76±0.91[b]	12.46±0.18[b]	12.61±0.49[b]	12.75±0.76[b]
2-Hexylfuran	15.725	1318	1323	0.65±0.11[a]	0.10±0.02[b]	0.12±0.02[b]	0.11±0.02[b]	0.12±0.02[b]
2-Heptylfuran	18.76	1419	1429	0.79±0.04[a]	0.15±0.03[c]	0.25±0.05[b]	0.17±0.03[c]	0.17±0.01[bc]
cis-Linalool oxide	18.91	1425	1425	6.43±0.32[a]	2.05±0.14[bc]	2.20±0.15[b]	1.87±0.16[c]	1.75±0.14[c]
trans-Linalool oxide	19.695	1453	1452	4.09±0.23[a]	1.67±0.18[b]	1.57±0.13[bc]	1.28±0.05[bc]	1.13±0.08[c]
**Others**								
Toluene	6.2	1033	1033	7.12±0.78[a]	2.56±0.14[b]	2.26±0.11[b]	2.43±0.31[b]	2.15±0.29[b]
1,3-dimethylbenzene	10.73	1167	1164	1.33±0.08[a]	1.30±0.03[a]	1.35±0.13[ab]	1.13±0.10[b]	1.25±0.10[ab]
N,N-Dimethylformamide	15.23	1301		0.26±0.03[a]	0.11±0.03[b]	0.09±0.03[b]	0.12±0.03[b]	0.15±0.03[b]
2-Methyl butyric Acid	24.765	1650	1652	4.60±0.59[a]	0.99±0.15[b]	1.46±0.28[b]	1.31±0.11[b]	1.07±0.24[b]
Pentanoic acid	26.385	1718	1719	6.13±0.84[a]	3.17±0.26[b]	2.44±0.19[bc]	2.03±0.26[c]	1.86±0.10[c]
Hexanoic acid	28.88	1825	1825	52.66±1.70[a]	15.68±1.34[b]	14.57±0.92[bc]	10.65±1.44[c]	10.30±0.13[c]
Nonanoic acid	35.665	2137	2137	12.17±2.52[a]	1.06±0.25[b]	1.43±0.24[b]	1.16±0.26[b]	0.39±0.08[b]

[1] RI_{cal}, the experimental Kovat's retention index calculated based on a DB-WAX capillary column.

[2] RI_{ref}, the Kovats' retention index information obtained from the NIST Chemistry WebBook database (https://webbook.nist.gov/chemistry/name-ser/).

Data were presented as mean ± standard deviation. For each SS treatment time, values with different superscript letters in rows were significantly different (p<0.05).
